# Nurse-Led Preoperative Education for Elective Surgery: Patient Satisfaction and Recall in a Mixed-Method Study

**DOI:** 10.3390/healthcare13222951

**Published:** 2025-11-18

**Authors:** Fatmah Jabr Alsolami

**Affiliations:** Faculty of Nursing, Umm Al-Qura University, Makkah City 24382, Saudi Arabia; fjsolami@uqu.edu.sa

**Keywords:** patient education, preoperative care, recall, surgery, satisfaction, quality of care

## Abstract

**Background**: Preoperative education is important in enhancing patients’ preparation and improving outcomes during recovery after surgery. However, there are limited studies in Saudi Arabia concerning levels of satisfaction and the recall of preoperative education among patients undergoing elective surgeries. **Aim**: The aim of this project was to examine patient satisfaction and recall of preoperative education for elective surgeries. **Methodology**: An explanatory sequential mixed-method study was carried out at one of the large governmental tertiary hospitals in the Western Region of Saudi Arabia. Data were collected from a total sample of 167 patients. Quantitative data were collected using a structured questionnaire, while interviews were carried out to collect qualitative data. Descriptive, inferential and thematic analyses were utilised for data analysis. **Results**: The quantitative results revealed high patient satisfaction with preoperative education (60%) and good levels of recall for preoperative education (45%). A moderate positive correlation between patient satisfaction and recall (r = 0.56; *p* < 0.01) was reported. A regression analysis revealed that age, level of education and surgery specialty predicted both satisfaction and recall. Four major themes (satisfaction with preoperative education, recall of preoperative education, effect of preoperative education on anxiety and preparedness and improvement suggestions) emerged from the qualitative analysis. **Conclusions**: This study established that patients under elective surgeries were satisfied with their preoperative education and could recall information provided in such educational programs. However, the findings also reinforced the need for follow-up communication after surgery to help improve recall and adherence to postoperative care instructions.

## 1. Introduction

The term ‘elective surgeries’ refers to procedures that are scheduled and performed on patients not when they are experiencing an emergency but rather to enhance their quality of life or address certain health issues [1]. These surgeries differ from urgent surgeries because the former allow patients and healthcare professionals time to prepare and organise themselves. Despite their predictable nature, elective surgeries involve certain challenges and risks. To realise success in such surgeries, it is necessary to focus on both the technical aspects of the operation and the management of patients. Preoperative education is a pillar of basic information that patients are intended to receive, and it entails a description of the surgical process and follow-up, the control of potential complications and an after-care process [2,3]. This education is crucial because it exposes patients to various facts about the preoperative, intraoperative and postoperative surgical periods [4].

Education before an operation is important in enhancing preparation amongst patients, reducing levels of anxiety and improving outcomes during recovery [5]. It is an obligatory tool that ensures that the patient is aware of the basic facts of the surgery, foreseeable complications and how to handle the required post-treatment [6]. For elective surgeries, the preoperative education provided should be effective, comprehensive, and straightforward [7]. Preoperative education is linked to several clinical outcomes, including a reduced recovery duration, the reduction in postoperative complications and patient satisfaction [4,8]. The objective of preoperative education is to educate patients about the expectations for surgical process and provide knowledge about how to address health management during the recovery process [9].

Quality of care includes patient satisfaction, which is the core element of healthcare [10,11]. The satisfaction of patients with preoperative education is defined as a vital indicator of information quality. It is emphasised that the provision of detailed and comprehensible information increases satisfaction and that it also increases confidence and alleviates tensions during the surgical process [12]. A recent study on preoperative education revealed that patients who had received a sufficient amount of education regarding their surgeries were relatively more satisfied with their experience and more prepared for their hospital experience and that there was a positive change in terms of their recovery [13]. On the other hand, providing inadequate preoperative information or ineffective information discourse may cause confusion, frustration, and disappointment, which affect patient satisfaction negatively and result in negative outcomes during the recovery process [14]. Moreover, the inability to satisfy the patient with preoperative education can lead to increased levels of anxiety and stress, which can also complicate the postoperative process, increase the lengths of hospital stays, and worsen the recovery process [15].

The memory of preoperative education also plays a significant role in defining the success of elective surgeries [15]. Despite the popularity of equipping patients with educational materials, the way in which patients remember and recall the educational materials is significant, as it determines how they follow through on the care instructions postoperatively, particularly regarding pain relief and wound management [16]. Most patients who have the potential to memorise the information related to their surgery and post-surgery procedure showed fewer complications and improved satisfaction. Nevertheless, recent studies have found that despite providing patients with high levels of preoperative education, they experience problems recalling the necessary information for multiple reasons, such as cognitive overload, anxiety, and the time gap between teaching and surgery [17,18]. This shows that there is a need to carry out successive education and utilise measures that can enhance the retention of the information.

Patient education and its effects on the results of a surgical intervention have been examined [19,20]. Nevertheless, there is a research gap regarding patient satisfaction and the retention of preoperative education over the course of elective surgical procedures that remains to be filled. The available literature on the topic has largely focused on general education on healthcare, with minimal investigation of satisfaction with and the recollection of preoperative education for elective surgical procedures. Alhajjaj et al. (2024) mention the educational needs of patients undergoing surgery but do not mention the elements of satisfaction or recall [20]. AlFaifi et al. (2023) also point out the need to offer systematic preoperative education but fail to concentrate on patient recall and satisfaction [19].

Preoperative patient education is a core nursing competency with consistent aims optimizing patient preparedness and safety, and commonly used delivery approaches, including nurse-led counseling supported by written or digital materials. The existing literature in pays limited attention to the role of nursing-led patient education and its effectiveness, especially during the preoperative phase.

Therefore, attempts to determine the impact of pre-operative education on patient satisfaction and recall, particularly for elective surgeries, which are often decided on short notice, should include assessments of whether brief preoperative education provided by nurses improves patient’s satisfaction.

To address this gap, this study was intended to concentrate on the satisfaction rate of patients with preoperative education provided by nurses as well as the process of recalling the information provided to the patients before elective surgeries in the Saudi Arabian setting. Using mixed-method research, this study provides insight into the effectiveness of preoperative educational interventions in terms of enhancing satisfaction and the recollection of information.

## 2. Material and Methods

### 2.1. Design

In this study, we employed an explanatory sequential mixed-method design. The first phase involved a structured questionnaire [12], which was followed by the second phase, in which semi-structured interviews with the same participants were used to elaborate on and explain the results obtained during the first phase. Integration occurred through connecting the results obtained from the first phase to inform the second phase’s sampling and questions, derive interview prompts from survey findings and create joint displays that juxtaposed the quantitative results with qualitative themes.

Quantitative and qualitative methods were employed to provide a comprehensive understanding of satisfaction with and the recall of preoperative education in elective surgeries. The quantitative aspect consisted of measuring the satisfaction rate of patients and the extent to which they recall the educational information provided during the preoperative period using standard measurement scales. The qualitative component incorporated detailed interviews to learn about the experiences with, perceptions of and problems with preoperative education among the patients and thus gain a better understanding of what contributes to significant satisfaction and recall. The mixed-methods design was the most appropriate study design to use in the research project because it allowed for the integration of the information from both approaches to enhance the extent and validity of the outcome findings [21,22].

### 2.2. Setting

This study was conducted in a Ministry of Health affiliated tertiary care hospital that provides comprehensive elective surgical service in Saudi Arabia. The facility serve a board adult population and hosts multidisciplinary medical and surgical teams with established pre-admission assessments, day-surgery, and inpatient pathway for elective procedures.

### 2.3. Participants

Eligible participants were adult patients ≥18 years scheduled for elective surgery 24–72 h before data collection, were in stable condition and reported no postoperative pain and who received a pre-operative education by nurses. Such patients were included to optimise the accuracy of postoperative recall. These patients were selected at their inclusion into the education program of the hospital, which is aimed at preparing patients for surgery. Patients who underwent emergency surgeries, were not able to grasp the educational resources, or had cognitive disorders affecting their recall of information were excluded.

### 2.4. Sampling

The quantitative sampling method chosen was convenience sampling. The patients involved in the sampling were potential participants who could conveniently volunteer to participate in this study. During their preoperative consultations, they were encouraged to take part in this study if they met the inclusion criteria. Convenience sample was used because of its convenience and effectiveness in sampling a large number of patients within a relatively short period of time.

The qualitative subsample was drawn from the survey cohort using purposive, maximum-variation sampling. During the questionnaire, participants could agree to a follow-up interview by indicating their willingness to be recontacted. Only those who provided permission were eligible for the qualitative phase. From this pool, we purposively selected interviews to maximise variation across surgical specialties, levels of satisfaction with pre-operative education, and key demographic characteristics, ensuring a range of experiences and perspectives. Eligible individuals were subsequently approached, asked to reconsent to the interview, and scheduled at a convenient time.

### 2.5. Sample Size

The quantitative aspect of this study involved applying a statistical approach and power analysis to determine the size of the sample. The parameters considered during the power analysis were as follows: A confidence level of 95% was used (alpha = 0.05), the presumed power was 80% (1–0.5 = 0.80), and a medium effect size was presumed to equal 0.5. The above parameters led to power analysis indicating the inclusion of 152 participants. This was an appropriate sample size that could help to find statistically important variability in patient satisfaction and preoperative education recall across various demographics and obtain reliable and meaningful results. A sample size of 15 patients was established for the qualitative component. This figure was selected to ensure the interviews yielded rich, in-depth data that could be organised into themes. This sample range was considered sufficient to retain a diverse range of perspectives and make the interviews manageable regarding information collection and analysis.

### 2.6. Data Collection

#### 2.6.1. Quantitative Data Collection

A structured questionnaire was used for the collection of quantitative data. The questionnaire comprised three sections: sociodemographic information, patient satisfaction, and patient education questionnaires.

***Sociodemographic Information:*** This section of the questionnaire comprised questions regarding respondent age, sex, educational level, occupation, marital status, and surgical specialty.

***Patient Satisfaction Questions:*** Patient satisfaction with preoperative education was measured using a questionnaire adopted from a previous study carried out in Ethiopia [1]. It has 24 items scored on a 5-point Likert scale, which ranges from ‘Very Satisfied’ (5 points) to ‘Very Dissatisfied’ (1 point). Total scores range from 24 to 120, and the interpretation of the scores is based on the following categories: low satisfaction (24–47), moderate satisfaction (48–72), and high satisfaction (73–120). The reliability of the questionnaire used in current study was established to be 0.89, indicating it is a reliable measure of patient satisfaction with preoperative education.

***Patient Education Questionnaire:*** The recall of preoperative education was measured using the patient education questionnaire adopted from Deressa et al. (2022) [1]. The questionnaire has 16 items, which were scored using a dichotomous key (correct or incorrect). A correct option is allocated one point, and zero points are awarded for an incorrect option. Total scores range between 0 and 16. The scores were categorised as indicating low recall (0–5), moderate recall (6–10), or high recall level (11–16). The reliability of the questionnaire in the current study was established to be 0.79, indicating it is a reliable measure of patients’ recall of preoperative education.

The scales were translated into Arabic language and reviewed for content validity by two bilingual academic nursing faculty to ensure the linguistic accuracy and cultural appropriateness. Minor wording adjustments were made to improve the clarity. The field-tested the Arabic version was on 60 nursing students prior the conduction of this study to ensure the internal consistency that was good (Cronbach’s Ⴛ = 0.86), supporting the reliability of the adopted measures in this population.

#### 2.6.2. Qualitative Data Collection

Following the quantitative survey, we conducted in-depth, semi-structured interviews with a purposively selected subsample of 15 survey respondents who reported their willingness to participate in the interview. These patients were selected based on their experiences regarding the preoperative education program, and we included a wide distribution of perspectives concerning patient satisfaction and the recall of preoperative education. Consistent with an explanatory sequential design, the interview guide was inspired by the findings of the survey results, allowing the participants to elaborate on their experiences with the education received. The semi-structured interview guide included open-ended questions, giving the participants a chance to expand on their thoughts and experiences. The interviews were conducted by a qualified, independent interviewer with formal experience in qualitative data collection and analysis, not involved in participants’ clinical care, using a standardised guide and confidentiality assurance to minimize positionality-related influence.

### 2.7. Data Analysis

#### 2.7.1. Quantitative Data Analysis

The outcomes of this study were interpreted with the use of inferential and descriptive statistics. The quantitative data were calculated using descriptive statistics to indicate the characteristics of the sample and the key variables of interest. Means and standard deviations were calculated to provide a description of how the scores are distributed. Pearson correlation analysis was used to comprehend the relationship between patient satisfaction and the recall of preoperative education. A multiple regression analysis was undertaken to establish the independent variables associated with patient satisfaction and the recall of preoperative training.

#### 2.7.2. Qualitative Data Analysis

The transcription of patient responses during the interviews was based on a verbatim technique often used in qualitative data analysis. This was accomplished through thematic analysis and a six-step framework [23], which was chosen due to its proven validity and reliability in other studies of a similar nature and because the guidelines it offers are sufficiently flexible when conducting qualitative studies [24]. The first step was to become acquainted with the data by reading the transcribed data multiple times. This was followed by preliminary coding to identify the key phrases and ideas. Such codes were subsequently grouped into major themes related to patient satisfaction and recall. After the completion of the first coding process, the themes were read and adjusted according to the data so that they would be more relevant to the data. The final themes were interpreted regarding the purpose of this study, and direct quotations of the participants were given to illustrate the points intentionally. Integrating both forms of data allowed us to examine how preoperative education affects both patient satisfaction and recall and to define potential areas of improvement in terms of educational practices.

### 2.8. Ethical Considerations

Ethical approval was obtained from the Nursing Scientific Research Committee from the Faculty of Nursing in Umm Al-Qura University on 15 May 2025 (Approval No. NSRC-15052025-84). Upon obtaining the ethical approval, the hospital administration was notified and granted the researcher access to eligible patients in the surgical unit. The researcher also hold an adjunct clinical appointment at the same hospital which facilitate the logistics for the conduction of this study. The questionnaire included participant information form and embedded informed consent form. Participants were required to read the information form and provide their consent by ticking the designated box before commencing the questionnaire. The same consent information was reiterated and reconfirmed before the interviews for the participants to complete before starting the qualitative data collection process. Participation was voluntary, and patients had the right to withdraw at any time without any impact on their care. The data were anonymised/coded and stored on the university’s password-protected OneDrive, and the findings are reported in aggregate with no identifying information.

### 2.9. Trustworthiness

To enhance this study’s rigor, multiple procedures were used to safeguard the credibility and validity of the results. Credibility was supported through prolonged engagement, as the interviewers spent ample time with the participants to gain a nuanced understanding of their experiences. Member checking was conducted after the interviews, as the participants reviewed their key findings to verify their accuracy and alignment with their experiences. Transferability was addressed by offering rich, detailed description of the context, sample, and procedures, enabling readers to judge the applicability of the findings to comparable settings. In addition, verbatim participant quotations were included to authentically convey their perspectives.

## 3. Results

### 3.1. Quantitative Results

This section provides a detailed analysis of the quantitative data relevant to this study on patient satisfaction with and recall of preoperative education regarding elective surgical procedures undertaken in a governmental tertiary hospital in the Western Region of Saudi Arabia.

***Demographic Characteristics of the Respondents.*** Table 1 provides a summary of the demographics of the subjects who participated in this study, including age, sex, level of education, occupation and surgery specialty. The findings revealed that majority of the respondents were female (52%), with the majority of them from the 31–45 age group (65%), and had a tertiary education (42%). General surgeries constituted the majority of surgeries conducted (62%).

***Frequency of Responses: Patient Satisfaction with Preoperative Education.*** The 24-item patient satisfaction questionnaire was used to assess various aspects of preoperative education. The frequency distributions for each item are summarised in Table 2. Most responses to the satisfaction questions indicate high satisfaction levels, with mean scores ranging from 4.0 to 4.2. Items related to the clarity of information, the availability of physicians, and nurses’ responsiveness received high satisfaction ratings. In some areas, such as waiting times and food/water supply, a small percentage of respondents were dissatisfied (5–6%), indicating that these areas may require further attention. Overall, the findings suggest that the preoperative education program was well received, with patients being generally satisfied with the quality of care, the clarity of the information, and the timeliness of the educational materials provided.

***Overall Patient Satisfaction Level.*** The overall patient satisfaction level was determined by summing the responses to the 24 items on the satisfaction questionnaire. The total satisfaction score ranged from 24 to 120, with higher scores indicating higher levels of satisfaction. The results were categorised as indicating low satisfaction (24–47), moderate satisfaction (48–72), and high satisfaction (73–120) as shown in Table 3. The overall satisfaction levels indicate that most patients (60%) reported high satisfaction with the preoperative education they received. Twenty-five percent of participants were moderately satisfied, and 15% reported low satisfaction, suggesting that there is room for improvement, particularly in terms of addressing the concerns of the minority who were dissatisfied. The relatively high satisfaction rate reflects the overall effectiveness of the preoperative education program, though areas of improvement should be targeted to address the needs of dissatisfied patients.

***Frequency of Responses: Recall of Preoperative Education.*** The recall of preoperative education questionnaire consists of 16 items. Participants responded with ‘Yes’ or ‘No’ to indicate whether they remembered specific pieces of information provided during preoperative education (Table 4). Most patients remembered key aspects of preoperative education. Over 85% of participants reported remembering critical items such as the type of surgery, the risks involved, and postoperative pain management. Some areas, such as the discontinuation of anticoagulant drugs (19% did not recall) and postoperative exercises (18% did not recall), had lower recall rates, suggesting that these topics could benefit from further emphasis or follow-up communication. A few other areas, such as treatment options other than surgery (20% did not recall) and the need for postoperative employment status information (17% did not recall) also had lower recall rates. This highlights the need for more targeted reinforcement or additional educational tools to aid recall.

***Overall Recall of Preoperative Education.*** The overall recall of preoperative education was determined by summing the responses to the 16 items on the recall questionnaire. The recall scores ranged from 0 to 16, with higher scores indicating better recall. The results were categorised into low recall (0–5), moderate recall (6–10), and high recall (11–16). The recall of preoperative education shows that 45% of patients had high recall, indicating that they remembered most or all the information provided. Forty percent of patients had moderate recall, while 15% had low recall, indicating that a portion of patients struggled to retain the key information as summarised in Table 5. While the overall recall rate is quite good, these results suggest that further strategies, such as follow-up communication or multimedia reinforcement, may be beneficial in terms of enhancing retention, particularly for patients who had low recall scores.

***Correlation Analysis.*** The Pearson correlation between patient satisfaction and recall was calculated to assess the strength of this relationship as shown in Table 6. There is a moderate positive correlation between patient satisfaction and recall (r = 0.56; *p* < 0.01), indicating that the patients who were more satisfied with their preoperative education were also more likely to remember the information provided.

***Regression Analysis.*** A multiple regression analysis with predictor variables included as model factors was applied to explore aspects of patient satisfaction and recollection. It was found that age, level of education, and surgery specialty were important predictors of both satisfaction and recall. Furthermore, level of education and type of surgery were factors that strongly determined recall, as general surgery patients had better recall as compared to patients who underwent other kinds of surgery (Table 7). These results support the necessity of individualised preoperative education based on patient profile and surgical situation.

### 3.2. Qualitative Results

A total of 15 anonymous interview recordings were transcribed verbatim by the researcher and a research assistant who was qualified to work with qualitative interview data. Then, the accuracy was verified by the researcher (Table 8). The interviews transcripts were imported into NVivo 12 for analysis. This analysis identified four themes and subthemes (Table 9). Each theme and subtheme represents how patients experienced preoperative education, including their satisfaction, recall and suggestions regarding how to improve it. Data saturation was set in advance as the absence of new codes in two back-to-back interviews. Data saturation defined a priori as (a) the absence of new codes across two consecutive interviews, and (b) subsequent codebook stability over the next two. Empirically, no new codes emerged after interview 12, and the codebook remained stable through interview 13 to 15.

### 3.3. Theme 1: Satisfaction with Preoperative Education

#### 3.3.1. Subtheme 1.1: The Clarity and Reassurance of Education

Most patients indicated that they were satisfied with the clarity and level of information they were given preoperatively. They noted that their understanding of what to expect during the surgery and afterwards made them feel confident and well prepared.

‘The procedural description regarding the operation was rather clear. I could tell what was going to happen, and it really put me at ease’

(Q1)

‘I was quite well informed about all the timing, risks, and post-surgery experiences. It gave me a sense of control’

(Q5)

This indicates that effective communication about what to expect during and after surgery through clear and comprehensive information was instrumental in alleviating anxiety.

#### 3.3.2. Subtheme 1.2: Educational Format Satisfaction

A significant number of patients strongly favoured multimodal learning, in which written text and visual aids were provided along with oral instructions.

‘The pamphlet was quite helpful. I would read it through and through so that I would not forget’

(Q2)

‘The video was useful, but I believe, even more, I would prefer having a printed guide to refer to afterwards’

(Q7)

This indicates that the patient can memorise information when they are able to look through visual and written material at their leisure. The use of multimodal resources in the form of pamphlets, videos, and illustrations ensured that patients could revisit important messages in a way that verbal instructions did not allow. This theme emphasises the need to complement verbal learning with written and visual materials, particularly in patients who may experience difficulties retaining information during stressful events.

### 3.4. Theme 2: Recall of Preoperative Education

#### 3.4.1. Subtheme 2.1: Excellent Recollection of Surgical Process and Preparation

The reporting rate for critical information on surgical preparation was generally high amongst the patients. Instructions related to fasting, medication changes and surgical timing were easily recallable for most patients.

‘I recall the date and time to stop eating and drinking and how to prepare for anesthesia. That was clear’

(Q4)

‘I would simply be able to tell when my surgery would be and what I would need to do in the morning. It made me feel in control’

(Q10)

This suggests that where preoperative education is performed regarding crucial preparation areas, such as surgery timing or medication, patients retain most of this knowledge. The fact that these essential details are well recalled indicates the effectiveness of the education in terms of the preparedness of patients for the surgical process, thus potentially reducing anxiety and enhancing feelings of confidence regarding being able to control the surgery.

#### 3.4.2. Subtheme 2.2: Weaknesses in Postoperative Instruction Recollection

Many patients remembered being prepared before the operation, but it was more difficult for them to remember postoperative instructions. Patients found it especially difficult to memorise information about pain management, wound care and activity restrictions.

‘I knew what to expect during surgery, but afterwards, I was unclear about what to do with my wound or how I could manage the pain. I had to ask again’

(Q15)

‘I recall being told about the pain management, but during the moment when I needed it, the specifics regarding the amount and frequency slipped my mind’

(Q6)

This indicates that the instructions on post-surgery treatment were not as readily held in the memory as the preoperative factual information, perhaps because of stress or exhaustion post-surgery. These may also include an increase in postoperative education, which can be enhanced by postoperative reminders or visuals aid that reminds patients of the key facts they should know regarding their postoperative period.

### 3.5. Theme 3: The Effect of Preoperative Education on Anxiety and Preparedness

#### Subtheme 3.1: Anxiety Reduction and Sense of Control Through Pre-Op Education

The analysis identified the anti-anxiety aspect of preoperative education. A significant portion of patients reported that they experienced a better level of preparation and a reduction in anxiety due to the availability of detailed information about the surgery and recovery

‘I was very concerned about what was unclear until the session when they clarified everything, and I felt so much better. It was comforting knowing what to expect’

(Q9)

‘I felt more comfortable because I knew everything. It reassured me about the doctors and my capacity to cope with the recovery process’

(Q12)

These quotes indicate that one important factor was preoperative education that alleviated pre-surgery anxiety and boosted confidence, which could have led patients to report more positive attitudes towards their surgical experience. Consequently, patients felt more in control, which minimised the stress related to the surgical process.

### 3.6. Theme 4: Improvement Suggestions

#### 3.6.1. Subtheme 4.1: Tailored, Condition-, and Procedure-Specific Education

Some patients indicated that preoperative education could be more individualised based on their health conditions and the surgical interventions they were to undergo.

‘I would have preferred that they discussed more about my illnesses and how they would impact the surgery. As an example, I have diabetes, and I was unsure about how it would affect my recovery’

(Q8)

‘They provided an overview of what to expect, but I would have appreciated hearing further details about my surgery and the warning signs of complications’

(Q13)

This feedback suggests that education should be more individualised to meet individual patient needs, as opposed to having a general perspective. Personalised learning may allow the patient to feel that they are well equipped with knowledge about their specific surgery and recovery experience, enhancing satisfaction and memory.

#### 3.6.2. Subtheme 4.2: Post-Operative Reinforcement and Support

Follow-up communication, which may include reminder messages about postoperative care and recovery procedures, was proposed by numerous patients.

‘I could have had a follow-up call a couple of days after the surgery to remind me about the next steps. I felt tired and overwhelmed’

(Q7)

‘The follow-ups or a reminder/check-in after the surgery would have motivated me to stay on course with my recovery, particularly wound care and pain management’

(Q5, Q4)

This underscores the importance of post-op follow-up in reinforcing important recovery teaching and making patients feel supported during the healing process. Post-surgery support may help boost patient confidence and mitigate the risk of complications due to inadvertently not following instructions.

### 3.7. Mixed-Methods Integration

Using an explanatory sequential design, we first analysed the survey results to identify the quantitative patterns (e.g., satisfaction-recall correlation and association with age, education, and surgical specialty). Then, we conducted interviews to explain the patterns from the patient perspective. Table 10 presents a mixed-methods joint display that links each quantitative result to the corresponding qualitative themes and example quotations, and distills these into concrete, practice-oriented implications for nurse-led pre-operative education.

## 4. Discussion

In this study, we examined patient satisfaction with and recall of preoperative education for elective surgeries. The quantitative results revealed that majority of the respondents (60%) had a high level of satisfaction with preoperative education. These findings align with previous studies that also reported that their patients had high satisfaction levels with preoperative education [12,25]. This study emphasised that appropriate and detailed preoperative information can diminish preoperative anxiety and help to develop sense of control and confidence among patients. The patients in the present study were satisfied with the clarity of the information presented to them regarding their surgeries, potential complications, and the process of recovery after the surgery. These findings support those of other studies that have shown that preoperative education contributes to better psychological outcomes and easy recovery [2,4]. However, the satisfaction level distribution, with 25% of patients having moderate satisfaction and 15% reporting a low satisfaction level, shows that although the educational process was satisfactorily received, patient experience varied. Empirical research has also pointed out that patients can be dissatisfied with preoperative education because of inadequate communication, excess information, or a failure to accommodate individual requirements [26]. The 15% of patients who expressed low satisfaction could be offered more customised or personal educational interventions that directly respond to their concerns and expectations.

In terms of the recall of preoperative education, the results demonstrated a good recall level, with 45% of the patients reporting such. The patients reported that they could remember facts related to the time when they underwent surgery and changes in medication use, among others. This aligns with the findings of other researches, who have shown that their participants could remember key preoperative education data [18,26]. These findings suggest that the educational program conducted in the hospital was effective in terms of ensuring that the patients were given and remembered the most important content regarding their operations. Conversely, the results indicate that patients reflected more poorly on post-surgery advice, such as post-surgery pain management, wound care and limited activity. This finding confirms the concerns expressed by Garfinkel et al. (2020), who also point out that patients have problems with recollecting information that is more detailed and more relevant to preoperative education, particularly when it refers to postoperative care [17]. The low recall level in these respects can be associated with cognitive load and stress, which may affect memory retention, anesthesia, or painkillers’ effect [16]. Patients are often unable to assimilate and remember complex information. Such results indicate that though the preoperative education program is effective, simple postoperative educational steps should be provided. It is possible that adding follow-up communication, which would involve reminder calls or written materials, would have a positive impact on retention, thus making patients recall significant information regarding elective surgeries.

The correlation results revealed a moderate positive correlation between satisfaction and the recall of information. This correlation indicates that more satisfied patients had a higher rate of remembering information. Age, level of education and type of surgery were found to be predictive factors for satisfaction as well as recall. This observation highlights the need to customise the educational material for each specific patient and, accordingly, present them with information that is formatted and delivered at a level corresponding to their cognitive capacity [12]. There was an increase in the recall rates in patients undergoing general surgery as opposed to orthopedic and urological surgeries. This may be attributed to the variation in the complexity and the amount of information given to these patients. General surgery patients may have been subjected to simpler instructions as compared to those facing orthopedic and urological surgeries, who had to process more complex or special instruction, which are more difficult to memorise.

The qualitative findings of the current study allow for insights into the experiences of patients exposed to preoperative education, specifically their satisfaction and recall. The qualitative findings revealed that patients were satisfied with their preoperative education. Satisfaction was associated with the clear presentation of information, which positively impacted their confidence and readiness level. In most cases, the respondents stated that their education was easy to understand and always reassuring and made them feel that they understood their surgeries. The current results are aligned with the findings of previous studies, which have reported that adequate information and communication about a surgical procedure and care after it helped decrease the preoperative level of anxiety and improve patient satisfaction [12,13]. The clearness of the information concerning the time of the surgery, the potential risks and postoperative life was also valued by patients. Such kinds of information are needed to prepare patients psychologically, as it has been proven that patients who are better informed can manage the stress and anxiety associated with surgery more efficiently than others [7,9].

The patients expressed satisfaction with the educational format of their preoperative education. The majority of patients found multimodal learning, written materials, visual aids, and verbal instructions to be particularly useful. The respondents observed that pamphlets, videos, and printed guides allowed them to review information at their convenience, which was essential in retention. This is in line with Molher et al. (2022) and Kim et al. (2021), who propose that patients are more likely to choose multimodal education as compared to other forms of education because it is more patient friendly, bypasses challenges related to differences in learning styles, and contributes to information recall [16,18].

The theme of the recall of preoperative education demonstrated that the patients typically recollected the major details of their preoperative education, including the kind of operation they were to undergo and the associated complications. This is line previous studies that demonstrate that patients are more likely to remember the most significant and indicative information, especially that associated with the surgical operation and the immediate preoperative environment [27,28]. However, other patients also reported that they had problems recollecting more complex postoperative information, such as pain treatment, wound treatment, and restricted action. This aligns with Garfinkel et al. (2020), who demonstrate that due to factors such as cognitive overload and stress, patients cannot discuss detailed postoperative care requirements [17]. In addition, the results highlighted the importance of preoperative education in the context of anxiety among patients. Moreover, the theme of suggestions for improvement outlined the importance of the individualised treatment of preoperative exposure to information about particular surgical processes. The identified findings are consistent with those reported in the healthcare literature that advocates for individualised care during surgery processes [29,30].

In this study, our qualitative results helped to explain the quantitative pattern. Patients described education that was clear, structured and reassuring, which align with the high satisfaction observed (=60%). They also emphasized that simple, time-bound pre-operative instructions (fasting time, medication adjustment, arrival logistics) were easy to remember, matching the higher recall for pre-op logistics seen in the survey, whereas detailed post-operative guidance (wound care, medication dose/interval, activity limits) was harder to retain, consistent with the overall recall (=45%). Preference for multimodal materials (brief nurse-led counseling plus a plain-language handouts and short videos) mirror the positive associations of education level with both satisfaction and recall, suggesting formats that support varied literacy needs. Patient requests for procedure-specific tailoring and a 48–72 h follow-up correspond to the observed differences by surgical specialty and point to practical steps to improve recall where it lagged. Together, the qualitative accounts provide concrete reasons for the quantitative findings and indicate actionable refinements teaching-back, tailored addenda, early follow-up to sustain satisfaction while increasing recall.

## 5. Conclusions

Findings of the current research demonstrated that preoperative education plays a vital role in enhancing satisfaction and the recall of information among candidates for elective surgeries. Patients reported excellent satisfaction levels and showed good retention of key information, such as the type of surgery and potential risks. However, many patients experienced difficulty recalling critical postoperative instructions, including those regarding pain management strategies, wound care, and activity restrictions.

From a nursing perspective, this highlights the need to strengthen preoperative education by placing greater emphasis on postoperative self-care instructions. Nurses should adopt patient-centred teaching methods, such as using simplified language, visual aids, written handouts, and teach-back techniques to ensure comprehension and retention. Additionally, reinforcement through follow-up teaching sessions, digital reminders, or involving family caregivers can improve recall and adherence. Nursing implications therefore include tailoring education to the patient’s level of understanding, prioritising high-risk information, and ensuring the continuity of education across the perioperative period. This approach can ultimately reduce complications, enhance recovery, and improve overall patient outcomes.

Therefore, to enhance the applicability, the results of qualitative themes can be translated into practice-oriented steps that focus on implementing a multimodal nurse-led education supported by a brief counselling, and incorporate a brief tech-back to confirm preoperative instructions. This can include providing procedure, condition-specific information and a simple post-operative checklist such as fasting times, medication adjustments and admission logistics followed by a postoperative follow-ups for education reinforcement within the 48–72 h after the surgery.

Although participants did not raise cultural or gender-specific influences including family role in decision-making and language or health literacy barriers, their absence in our study should not be interpreted as lack of relevance. It is likely reflects our sampling, the interview guide emphasis and the clinician-interviewer context. Future studies should purposely include patients with diverse language backgrounds and caregiver involvement. In practice, nurse-led education should offer language-concordant materials, invite a designated family member when appropriate and include low literacy versions to better capture and address culturally grounded needs.

This study has some limitations of using the convenience sampling of adult elective-surgery patients from a single tertiary hospital that may introduce selection bias and limit the generalisability of findings beyond similar clinical environment. Although this study has used convenience sample to ensure that eligible patients could practically participate and complete the questionnaire because immediate post-operative pain and anaesthesia effects can hinder participation and data quality, it is suggested that future studies should mitigate this by employing consecutive multi-site sampling to enhance its generalisability.

## 6. Relevance to Clinical Practice

Nurse-led preoperative education is a core competency in professional nursing practice. patients often report high and good recall of information provided before surgery. To optimize outcomes, educational interventions should be deliberately designed to address patients’ concerns both pre-and postoperatively, rather than focusing exclusively on the preoperative phase; structured, staged delivery across the perioperative pathway reinforces key messages and supports informed participation in care.

## Figures and Tables

**Table 1:** Demographic characteristics of participants (n = 152).

Characteristic	Frequency	Percentage (%)
**Sex**		
Male	73	48%
Female	79	52%
**Age**		
18–30	28	18%
31–45	99	65%
46+	25	17%
**Education Level**		
Secondary	56	37%
Tertiary	64	42%
Postgraduate	32	21%
**Occupation**		
Public Service	50	33%
Private Sector	40	26%
Other	62	41%
**Surgical Specialty**		
General Surgery	94	62%
Orthopedic	38	25%
Urology	20	13%

**Table 2:** Frequency of responses to patient satisfaction items (n = 152).

Satisfaction Item	Very Dissatisfied	Dissatisfied	Neutral	Satisfied	Very Satisfied	**Mean ± SD**
1. Adequacy of physicians’ and nurses’ answers to your questions?	3 (2%)	5 (3%)	20 (13%)	55 (36%)	69 (45%)	4.2 ± 0.9
2. Adequacy of physicians’ and nurses’ information about the nature of your problem or operation?	4 (3%)	7 (5%)	18 (12%)	60 (39%)	63 (41%)	4.1 ± 1.0
3. Adequacy of information provided about the potential complications that may occur during operation or treatment?	5 (3%)	8 (5%)	25 (16%)	57 (38%)	57 (38%)	4.0 ± 0.9
4. Waiting time to see the doctor?	6 (4%)	9 (6%)	29 (19%)	52 (34%)	56 (37%)	4.0 ± 1.0
5. Waiting time to provide laboratory specimens?	7 (5%)	10 (7%)	26 (17%)	53 (35%)	56 (37%)	4.0 ± 1.0
6. Waiting time to see the doctor after receiving lab results?	8 (5%)	10 (7%)	25 (16%)	54 (36%)	55 (37%)	4.0 ± 1.0
7. Nurses’ responsiveness to your call?	4 (3%)	6 (4%)	29 (19%)	57 (38%)	56 (37%)	4.1 ± 0.9
8. Nurses’ respect for you during communication?	3 (2%)	5 (3%)	27 (18%)	56 (37%)	61 (40%)	4.1 ± 0.9
9. Adequacy of information ward nurses provided to you about your health progress?	5 (3%)	7 (5%)	30 (20%)	54 (35%)	56 (37%)	4.0 ± 0.9
10. Adequacy of information provided by ward nurses about the importance of investigations?	6 (4%)	8 (5%)	29 (19%)	55 (36%)	54 (36%)	4.0 ± 0.9
11. Adequacy of information provided by ward nurses about the side effects of medications?	4 (3%)	7 (5%)	26 (17%)	59 (39%)	56 (37%)	4.1 ± 0.9
12. Adequacy of the time ward nurses spent with you during evaluation and treatment?	3 (2%)	5 (3%)	30 (20%)	53 (35%)	61 (40%)	4.1 ± 0.9
13. Adequacy of care given by ward nurses at night?	6 (4%)	8 (5%)	26 (17%)	56 (37%)	56 (37%)	4.0 ± 1.0
14. Responsibility of the physician for you?	4 (3%)	7 (5%)	25 (16%)	54 (35%)	62 (41%)	4.1 ± 0.9
15. The communication of the physicians with you in an understandable way?	4 (3%)	6 (4%)	29 (19%)	55 (36%)	58 (38%)	4.1 ± 0.9
16. The availability of the responsible physician when needed?	3 (2%)	5 (3%)	30 (20%)	56 (37%)	58 (38%)	4.1 ± 0.9
17. Did the physician show a caring attitude towards you?	4 (3%)	6 (4%)	29 (19%)	56 (37%)	57 (38%)	4.1 ± 0.9
18. Were you confident in the skill of physicians during treatment?	5 (3%)	7 (5%)	28 (18%)	54 (35%)	58 (38%)	4.1 ± 0.9
19. The cleanliness of the ward or beds?	3 (2%)	4 (3%)	25 (16%)	56 (37%)	64 (42%)	4.1 ± 0.9
20. The cleanliness of the bathroom?	4 (3%)	6 (4%)	27 (18%)	53 (35%)	62 (40%)	4.1 ± 0.9
21. The cleanliness of the latrines?	3 (2%)	5 (3%)	28 (18%)	54 (35%)	60 (39%)	4.1 ± 0.9
22. The adequacy of the food and water supply?	3 (2%)	6 (4%)	28 (18%)	55 (36%)	60 (39%)	4.1 ± 0.9
23. Accessibility of pharmacy and laboratory facilities to you?	4 (3%)	6 (4%)	29 (19%)	56 (37%)	57 (37%)	4.1 ± 0.9
24. The fairness of medication and investigation costs for you?	5 (3%)	6 (4%)	28 (18%)	56 (37%)	57 (37%)	4.1 ± 0.9

**Table 3:** Overall patient satisfaction level (n = 152).

Satisfaction Level	Frequency	Percentage (%)
Low (24–47)	23	15%
Moderate (48–72)	38	25%
High (73–120)	91	60%

**Table 4:** Frequency of responses to recall items (n = 152).

Recall Statement	No	Yes
1. Informed about type of surgery and surgical procedure	20	132
2. Informed about feeding (fasting time and duration, IV or tube feeding, care, postoperative first oral intake, and diet)	18	134
3. Informed about postoperative life (the effect of surgery on patient’s life)	17	135
4. Informed about treatment options other than surgery	23	129
5. Informed about day, time and duration of surgery	19	133
6. Informed about the risks of surgery	21	131
7. Informed about the type of anesthesia used	25	127
8. Informed about the need for discontinuation of anticoagulant drugs, if any	30	122
9. Informed about postoperative pain management, treatment, excretion, and individual care (bath, perineum care, etc.)	22	130
10. Informed about postoperative exercises (deep-breathing and cough exercises, range of motion, and the use of positive end-expiratory pressure) and activities of early mobilisation	28	124
11. Informed about wound care	21	131
12. Informed about drains/catheters and points to take into consideration	20	132
13. Informed about the post-anesthesia care unit, the intensive care process (if necessary), post-operative care, the recovery process, and patient involvement in care	23	129
14. Informed about postoperative employment status and changes in work/home life	25	127
15. Informed about postoperative treatment and follow-up/monitoring frequency	22	130
16. Informed about post-discharge home care practices and precautions	19	133

**Table 5:** Overall recall level for preoperative education (n = 152).

Recall Level	Frequency	Percentage (%)
Low (0–5)	23	15%
Moderate (6–10)	61	40%
High (11–16)	68	45%

**Table 6:** Correlation between patient satisfaction and recall.

Variable	Satisfaction	Recall
Satisfaction	1.000	0.56 **
Recall	0.56 **	1.000
Age	0.22 *	0.12
Education Level	0.32 **	0.28 **
Surgical Specialty	0.12	0.23 **

Note: * *p* < 0.05, ** *p* < 0.01.

**Table 7:** Multiple regression analysis for predictors of satisfaction and recall.

Predictor	β (Satisfaction)	*p*-Value (Satisfaction)	β (Recall)	*p*-Value (Recall)
**Age**	0.24	<0.01		
**Education Level**	0.18	<0.05	0.32	<0.01
**Surgical Specialty**			0.21	<0.05

**Table 8:** Characteristics of the interviewers.

Characteristic	N (%), Mean
**Sex**	
Male	7 (46.6%)
Female	8 (53.4%)
**Age**	
31–45	9 (60%)
46+	6 (40%)
**Surgical Specialty**	
General Surgery	7 (46.6%)
Orthopaedic	3 (20%)
Urology	5 (33.4%)

**Table 9:** Patients experience of their recall of preoperative education in elective surgeries.

Themes	Subthemes	Codes
**Theme 1: Satisfaction with Preoperative Education**	**1.1 The Clarity and Reassurance of Education**	Procedural Clarity Anticipatory Reassurance/Anxiety Reduction Sense of Control/Preparedness
	**1.2 Educational Format Satisfaction**	Multimodal Reinforcement Preference for Take-Home Written Aids
**Theme 2: Recall of Preoperative Education**	**2.1 Excellent Recollection of Surgical Process and Preparation**	Fasting and Medication Timing Recall Procedural Schedule and Morning-of Tasks
	**2.2 Weaknesses in Postoperative Instruction Recollection**	Wound Care Specifics Forgotten Analgesia Dosing and Frequency Slippage
**Theme 3: Effect of Preoperative Education on Anxiety and Preparedness**	**3.1 Anxiety Reduction and Sense of Control through Pre-Op Education**	Uncertainty Clarification Anticipatory Guidance Confidence in Care Team Coping Preparedness
**Theme 4: Patient Heterogeneity and Contextual Modifiers**	**4.1 Tailored, Condition- and Procedure-Specific Education**	Condition-Tailored Content Procedure-Specific Depth and Red Flags
	**4.2 Post-Operative Reinforcement and Support**	Follow-Up Reminders/Check-Ins Adherence Support and Motivation

**Table 10:** Mixed-methods joint display linking quantitative results to qualitative themes and practice implications.

Quantitative Results	Qualitative Themes Explaining the Pattern	Patient Insights	Integrated Interpretations	Practice Implications
**Satisfaction Level** Satisfaction: 1.000 Recall: 0.56	Theme 1.1 Clarity & reassurance; Theme 2.1 Strong recall for pre-op logistics	‘The procedural description regarding the operation was rather clear … it really put me at ease’ (Q1); ‘I would simply be able to tell when my surgery would be and what I would need to do in the morning’ (Q10)	Clear, structured counselling increase confidence (satisfaction) and anchors key facts (recall)	Keep a brief, scripted nurse-led core message; end every session with a 1–2 min teach-back.
**Recall** Satisfaction: 0.56 Recall: 1.000	Theme 1 & 3 (clarity reassurance) vs. Theme 2.2 (post-op recall gaps).	‘the specifics regarding the amount and frequency slipped my mind’ (Q6)	Patients feel satisfied even when post-op self-care details are not retained.	Do a day of surgery recap and a 48–72 follow up for information reinforcement.
**Education level** Satisfaction: Recall:	Theme 1.2 Preference for multimodal materials; Theme 2.2 Gaps in post-op recall	‘The pamphlet quite was helpful… so I wouldn’t forget’ (Q2)	Higher education may ease processing; multimodal materials mitigate literacy barriers.	Provide plain-language handouts, QR micro-video for all, chunk information, avoid jargon, use icons.
**Age** Satisfaction: 0.22 Recall: 0.12	Theme 3 Anxiety reduction & preparedness; Theme 2.2 Post-op recall weaker	‘They clarified everything and I felt so much better ‘ (Q9)	Older adults may value reassurance more; recall gains are smaller without reinforcement.	Add a calming micro-script (normalisation, breathing cue); supply a large-print checklist and involve a caregiver when possible.
**Surgical Specialty** Satisfaction: 0.12 Recall: 0.23	Theme 4.1 Tailored, procedure-specific education; Theme 2.2 Post-op details fade	‘I would have preferred that they discussed more about my illnesses and how they would impact the surgery’ (Q8)	Recall varies with procedure complexity and relevance of details.	Attach a procedure-specific addendum (e.g., wound drains, mobility limits, anticoagulation). Highlight 3 ‘must remember points’

## Data Availability

The data presented in this study are available upon request from the corresponding author. The data are not publicly available due to privacy or ethical restrictions.
